# The Electronic Frailty Index and Area Deprivation Index: Independent Constructs in Risk Stratification

**DOI:** 10.1093/geroni/igaa057.580

**Published:** 2020-12-16

**Authors:** Kathryn Callahan, Kristin Lenoir, Nicholas Pajewski

**Affiliations:** 1 Wake Forest School of Medicine, Winston-Salem, North Carolina, United States; 2 Wake Forest University Health Sciences, Winston-Salem, North Carolina, United States

## Abstract

Frailty and social determinants of health (SDOH) have been associated with mortality for older adults. Given time limitations, passive electronic tools such as the eFI and publicly available data such as the Area Deprivation Index (ADI) hold appeal for targeting limited resources to support at-risk older adults. Literature is conflicting with regards to the relationship between frailty and SDOH. A retrospective, observational cohort of adults 65+ (n=44,548) identified as part of the Wake Forest Baptist Health (WFBH) accountable care organization was used to evaluate the association between ADI, eFI, and mortality between 1/1/2019 and 1/1/2020. A cox proportional hazard model was fit, adjusting for age, sex, race, and weighted Charlson Comorbidity Index. Sources of mortality data include claims data, the EHR at WFBH, and NC Vital Statistics. Block-level geographic identifiers (GEOID) were extracted and used to merge ADI national percentiles (Neighborhood Atlas), derived from U.S. Census 5-year American Community Survey estimates, which incorporates 17 SDOH measures (e.g., income, education, housing, employment.) Frailty was calculated by the WFBH eFI. 9216 (20.7%) were frail by eFI (eFI>0.21) and 235 (0.5%) died. The interaction between ADI tertile and eFI category was not significant (p=0.78). Being frail was associated with poorer survival when compared to the fit group; HR= 1.94 (95% CI =1.23, 3.08; p<0.01.) Survival did not differ between the maximum deprivation tertile and the minimum tertile, HR=1.21 (95% CI=0.88-1.68, p=0.25). Frailty and SDOH may represent independent constructs in risk stratification for older adults. Future work will explore associations within healthcare utilization.

